# Early clinical outcomes and comparison between trans-PRK and PRK, regarding refractive outcome, wound healing, pain intensity and visual recovery time in a real‐world setup

**DOI:** 10.1186/s12886-021-01941-3

**Published:** 2021-04-16

**Authors:** Harald C. Gaeckle

**Affiliations:** Augenlaserzentrum Neu-Ulm, Edisonallee 19, 89231 Neu-Ulm, Germany

**Keywords:** Transepithelial PRK, Myopia, Visual acuity, Pain, Visual recovery, Wound healing

## Abstract

**Purpose:**

To compare early clinical outcomes of single-step transepithelial photorefractive keratectomy (tPRK) and photorefractive keratectomy (PRK) regarding refractive outcome, visual acuity, wound healing, pain intensity and visual recovery time.d.

**Methods:**

In this prospective clinical observational study 200 eyes of 100 consecutive patients with mild to moderate myopia with or without mild astigmatism were included. One hundred eyes each were either treated with StreamLight™ tPRK or PRK with the WaveLight® EX500 excimer laser. Visual acuity (Decimal) was assessed preoperatively and at day 4, 7 and 6 weeks postoperatively. Wound healing (hours between surgery and complete epithelial closure) was monitored at the slit lamp. At day 4, patients subjectively rated the maximum pain intensity within the last 4 days using a numerical pain rating scale (0–15).

**Results:**

Visual recovery was significantly faster in the tPRK group. At days 4 and 7, the mean monocular UCDVA was significantly better in the tPRK group than in the PRK group (*p* < 0.001). Four days after surgery 72 % of eyes in the tPRK group but no eye in the PRK had a UCDVA of 0.7 or better. At six weeks postoperatively, a UCDVA of 1.0 or better was achieved in both groups. Complete epithelial wound closure was achieved significantly faster in the tPRK group (*p* < 0.0001) and maximum pain level within the first 4 days after surgery was significantly lower in the tPRK group (*p* < 0.0001). No patient had lost a line of BCDVA and no complications or adverse effects were observed.

**Conclusions:**

According to our early clinical results, both treatments options appear to be safe and effective methods for the correction of low to moderate myopia with and without astigmatism. However, in our study, StreamLight™ tPRK offered faster visual recovery and epithelial healing and was associated with less pain compared to PRK. It can therefore be considered a good treatment option for patients who refuse or are not eligible for Femto-LASIK, but at the same time demand a faster and more comfortable recovery time than PRK can offer.

## Introduction

The introduction of single-step transepithelial photorefractive keratectomy (tPRK) procedures in recent years has led to a renaissance of surface ablation treatments in refractive surgery. For decades, photorefractive keratectomy (PRK) has been a well-established and safe surface ablation technique to correct low to moderate myopia and astigmatism [1, 2]. Moreover, it is also suitable for patients who reject or are not eligible for laser in situ keratomileusis (LASIK), e.g., due to thin corneas or subtle topographic irregularities [3, 4].

The first report on phototherapeutic keratectomy (PTK) on a human eye was in 1986 [5]. Today, there are several surface ablation techniques with a variety of methods for removal of the corneal epithelium prior to excimer laser treatment. This can either be performed using 20 % alcohol and a hockey knife, or without alcohol using a rotating brush. Another possibility is the use of an epikeratome for epithelial removal with the aim to place the epithelial layer back on the cornea after the excimer laser treatment (epi on LASEK), however, speed of wound healing using the epi on technique was not much different to the previous described techniques [6]. StreamLight™ or tPRK describes a technique where the corneal epithelium is removed using an excimer laser.

Corneal epithelial and stromal wound healing is a very complex process involving cytokines, glycoproteins (fibronectin) and plasminogen activator, finally leading to proliferation and migration of epithelial cells from the limbus and basal epithelial layer [7]. The ultraviolet radiation and thermal elevation from the laser, increases the production of oxygen free radicals and can lead to cytotoxic damage and apoptosis. To reduce this process of apoptosis, it is recommended to irrigate the cornea with ice-cold BSS before and after laser treatment like we always do. Theoretically, mechanical debridement of the corneal epithelium has been discussed to provoke stromal hyperplasia and opacification due to loss of anterior stromal keratinocytes and stromal dehydration [8].

In our practice, tPRK or PRK is the preferred method for all myopic patients with thin corneas under 500 μm. Also, patients with a history of epithelial basement dystrophies and patients with recurrent epithelial defects are recommended surface ablation methods in our practice, however, these patients were not included in this study. Collagen autoimmune disorders, cataract, glaucoma, uveitis, extreme dry eyes, or extreme ocular allergy are considered contraindications for Femtosecondlaser (FS)-Lasik as well as for surface ablations methods in our practice. Relative contraindications are poorly controlled Diabetes mellitus (possible decreased corneal sensation and delayed wound healing), history of Herpes Keratitis (possible reactivation of the HSV). Surface ablation methods are not recommended in our practice for hypermetropic patients and for patients with myopic spherical equivalent of more than 6 D and high cylinder over 3 D [9]. There seems to be some evidence that in patients with pigment dispersion glaucoma FS-Lasik might lead to a delayed healing and less predictable outcome [10].

Refractive outcomes after PRK are good and complications like corneal haze are rare [11, 12]. However, a long visual rehabilitation period and considerable postoperative pain deter many patients from opting for PRK [13, 14]. Since a few years “no-touch” single-step tPRK procedures are available from various manufacturers and have proven to be effective and safe [15–17]. StreamLight™ is a novel, one-step tPRK procedure in which the epithelium is first removed by phototherapeutic keratectomy (PTK) immediately followed by PRK in a single procedure using the Wavelight EX500 excimer laser. Due to newly calculated nomograms, size and location of the PTK treatment zone are automatically aligned with the PRK ablation profile and centration is only required once in StreamLight™ procedure. Moreover, a multidimensional eye tracker is active throughout the entire procedure.

The purpose of our prospective observational study was to evaluate the clinical outcomes of StreamLight™ tPRK in daily clinical practice and compare them to those of PRK regarding refractive outcome, visual acuity, wound healing, pain intensity and visual recovery time.

## Methods

### Study design and patients

This is a prospective clinical observational study including 200 eyes of 100 consecutive patients with mild to moderate myopia (− 2.0 D to -6.0 D spherical equivalent) and mild (0.0 D to -2.5 D) astigmatism who underwent PRK or tPRK at the Augenlaserzentrum Neu-Ulm, Germany between January and December 2019. After comprehensive information about risks and benefits of the two surgical techniques, patients were free to choose one of the two procedures. All patients provided a signed informed consent form for data collection, evaluation and publication. The research has been carried in accordance with the Declaration of Helsinki. All methods were performed in accordance with the relevant guidelines and regulations, although ethical approval was not required and deemed unnecessary according to national regulations of the Bavarian Medical Association (Bayerische Landesärztekammer (BLÄK) Ethikkommission (ethics committee), Mühlbaurstraße 16, 81,677 München), which states that study projects with anonymized data are not subject to consultation.

All patients qualified for surface ablation according to the German standards for surface ablation of the German Committee of Refractive Surgery (Kommission Refraktive Chirurgie) [18]. The Committee of Refractive Surgery (Kommission Refraktive Chirurgie, KRC), is a joint committee of the German Ophthalmological Society (Deutsche Ophthalmologische Gesellschaft, DOG) and the Professional Association of Ophthalmologists in Germany (Berufsverband der Augenärzte Deutschlands, BVA) and has issued generally applicable criteria for refractive surgical procedures with the aim of preventing unnecessary complications as much as possible (www.augeninfo.de/krc).

Exclusion criteria were unstable refraction, severe ocular surface disease, corneal epithelial pathology, keratoconus, any posterior segment pathology or any previous intraocular or corneal surgery. All patients were advised to discontinue contact lens wear for a minimum of 3 weeks prior to preoperative examination and treatment.

### Pre- and postoperative assessments

Preoperatively, all patients underwent a complete eye examination including uncorrected distance visual acuity (UCDVA) and best corrected distance visual acuity (BCDVA) assessment, manifest refraction, autokeratometry, intraocular pressure measurement, and slit lamp examination to evaluate the anterior segment and the fundus. Visual acuity (at 5 m, decimal) and manifest refraction measurements as well as corneal topography assessment by Allegro Topolyzer-Vario (WaveLight, Erlangen, Germany) and Scheimpflug tomography examination by Allegro Oculyzer ІІ (WaveLight, Erlangen, Germany) were performed by one experienced optometrist.

Postoperatively, slit lamp examinations were performed by one ophthalmologist at day 1, 2, 3, 4, 7, and 6 weeks after surgery. Monocular UCDVA was measured at day 4, 7 and 6 weeks postoperatively. Manifest refraction and binocular BCDVA were assessed at 6 weeks after surgery. Moreover, at day 4, patients retrospectively evaluated their subjective maximum pain intensity level within the first four days after surgery on a numeric pain rating scale (0–15) for each eye. This clinical pain rating scale was completed by the patients themselves. To facilitate the patients´ assessment, the categories “no pain” (0), “low” (1–5), “high” (6–10) or “very high” (11–15) were added to the visual analog scale. To assess wound healing during daily slit lamp examinations all patients were instructed to come back to the clinic the next morning (day 1) and for the next three days (day 2,3,4) at the same time of day that the surgery took place on day 0. If a closed epithelium was noted on the slit lamp, the hours since surgery were counted.

### Surgical procedures

All surgical procedures were performed bilaterally on the same day, with the right eye being treated first, by one single surgeon (HCG). The wavefront-optimized (WFO) ablation profile was planned using the standard planning software including WaveLight nomograms and was based on manifest refraction. In StreamLight™ procedures, a newly calculated shot matrix enables an equal ablation of the epithelium over the treatment zone (7mm for myopia and 9 mm for mixed myopic astigmatism).

In both groups standard wavefront optimization laser ablation profiles with a refractive ablation zone of 6.5 mm in all cases were applied and the use of Mitomycin C was avoided according to the recommendations of the German Committee of Refractive Surgery [18].

**Fig. 1:**
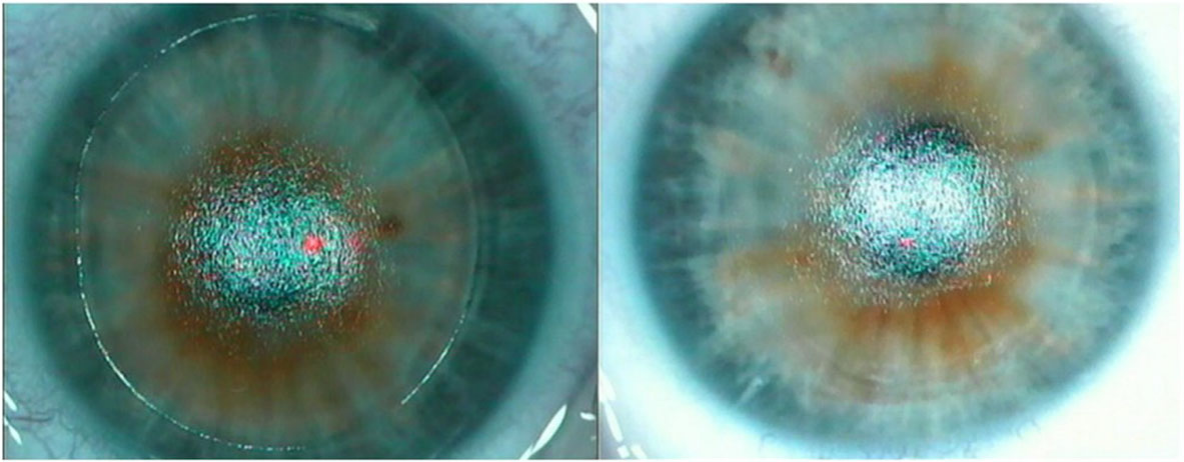
Typical picture of epithelial abrasion using tPRK with clearly visible margin of the ablation zone (**a**) and typical picture of epithelial abrasion using an Amoils brush (**b**)

The standard preoperative procedure for both procedures was the same. Topical proparacaine hydrochloride 0.5 % (Alcaine; Alcon Laboratories, Inc., Fort Worth, TX, USA) eye drops were instilled twice directly before surgery. In the PRK group, de-epithelialization was performed with a 9 mm rotating brush (Amoils Rotary Epithelial Scrubber, Innovative Excimer Solutions, Inc., Toronto, Canada), with the epithelial ablation zone determined by the diameter of the disposable brush head. Remaining epithelial cells were removed using a dry PVA eye spear. Subsequently, PRK laser ablation was carried out followed by 1 min of corneal and conjunctival cooling with ice-cold BSS administered with a syringe. In the tPRK group, conjunctiva and cornea were pre-cooled with ice-cold BSS from a syringe for 30 s. After removing excessive liquid from the conjunctiva and cornea with a dry PVA eye spear (max. 10 s.), de-epithelialization was performed using the StreamLight™ PTK mode for 22–30 s depending on preexisting astigmatism. StreamLight™ allows to individually determine the epithelial ablation depth between 45 and 65 μm after epithelial mapping. In our daily practice we usually work with an epithelial ablation depth of 55 μm. The Streamlight™ PTK mode is optimized not to induce any refractive shift. After the PTK mode, the cornea was checked and any remaining epithelial cells were removed mechanically with a dry PVA eye spear. Figure 1 shows typical intraoperative pictures of epithelial abrasion after both procedures prior to refractive excimer ablation. After an interruption of around 10 s to cool down the cornea, PRK laser ablation was applied followed by 1-minute cooling of the cornea and conjunctiva with ice-cold BSS administered with a syringe. Following both laser procedures preservative-free Ofloxacin and corticosteroid eye drops were instilled, a pre-cooled soft bandage contact lens (Acuvue ®; Johnson and Johnson Vision Care, Inc., Jacksonville, USA) was placed on the cornea and preservative-free eye drops containing 0,15 % sodium hyaluronate were applied. The soft bandage contact lens was removed 4 days after surgery in both groups. Patients were advised to continue three times daily with preservative-free corticosteroid eye drops for 4 weeks and to regularly use preservative-free 0,15 % sodium hyaluronate eye drops at least 5 times per day. All patients were allowed to take non-steroidal pain killers maximum 2 tablets of Ibuprofen 400 mg per day. Patients were also advised to put a wet ice-cold compress on the closed eyes to reduce pain.

### Statistical analysis

Results are expressed as mean ± standard deviation. Data were analyzed using Statview 5.01 software for Windows (Abacus Concepts, Inc., Berkeley, California). For determining statistical significance between both groups, the non-parametric Mann-Whitney U test and the t-Test for independent samples were performed (*p* < 0.05 considered statistically significant).

## Results

Demographic and baseline characteristics are displayed in Table 1. There was no statistically significant difference regarding demographic and baseline characteristics between the two groups (*p* > 0.05).

**Table 1:** Demographics and baseline characteristics

	**PRK** (*n*=100 eyes of 50 patients)			**tPRK** (*n*=100 eyes of 50 patients)		
**Age (years)**, mean ± SD	27.2 ± 3,3			27.7 ± 3.4		
**Gender,** n						
Male	16			20		
Female	34			30		
	Right eyes	Left eyes	**All eyes**	Right eyes	Left eyes	**All eyes**
**Refractive error (D),** mean ± SD,						
Sphere	-3.29 ± 1.20	-3.20 ± 1.20	**-3.24 ± 1.20**	-2.73 ± 0.98	-2.81 ± 1.02	**-2.77 ± 1.00**
Cylinder	-0.72 ± 0.67	-0.70 ± 0.65	**-0.71 ± 0.65**	0.65 ± 0.61	-0.64 ± 0.56	**-0.64 ± 0.58**
**UCDVA (decimal),** mean ± SD	0.21 ± 0.09	0.21 ± 0.09	**0.21 ± 0.09**	0.22 ± 0.08	0.22 ± 0.07	**0.22 ± 0.08**
**BCDVA (decimal),** mean ± SD	1.17 ± 0.18	1.18 ± 0.17	**1.17 ± 0.18**	1.10 ± 0.15	1.12 ± 0.15	**1.11 ± 0.15**

*BCDVA* best corrected distance visual acuity, *D* diopter, *UCDVA* uncorrected distance visual acuity, *PRK* photorefractive keratectomy, *tPRK* transepithelial photokeratectomy, *SE* spherical equivalent, *SD* standard deviation

**Fig. 2:**
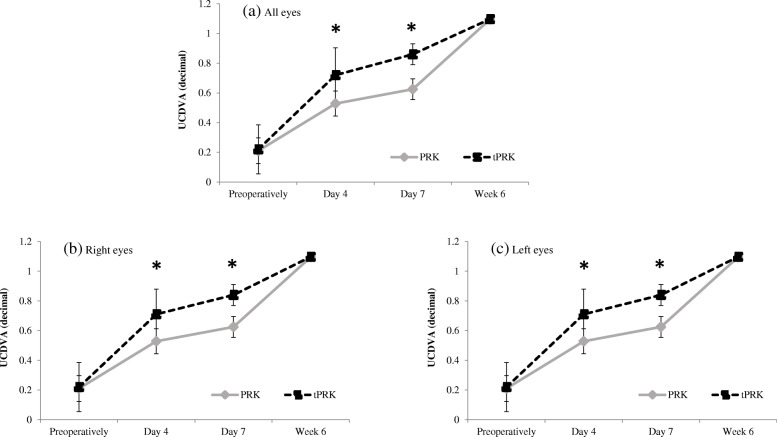
Mean monocular UCDVA (decimal) preoperatively and at 4, 7 days and six weeks postoperatively in both treatment groups groups for (**a**) all eyes, **b** right eyes, **c** left eyes. Error bars indicate SD for the mean. UCDVA at day 4 and day 7 was significantly better in the tPRK group (*p* < 0.001 same level of significance on both days)

**Fig. 3:**
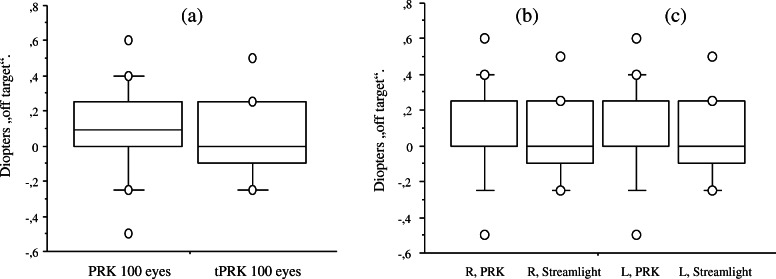
„Off target“ spherical equivalent at 6 weeks after surgery in both treatment groups for (**a**) all eyes, **b** right eyes, **c** left eyes (*p* > 0,05)

Postoperative results are summarized in Table 2. Visual recovery was achieved significantly faster in the tPRK group. At days 4 and 7, the mean monocular UCDVA (decimal) was significantly better in the tPRK group than in the PRK group (*p* < 0.001), while six weeks after surgery both groups had achieved a comparable mean UCDVA of better than 1.0 (*p* > 0.05) (Fig. 2a). No significant differences in re-epithelialization were observed between right and left eyes (Fig. 2b, c). Four days after surgery 72 % of eyes in the tPRK group had a UCDVA of 0.7 or more, while no eye in the PRK group reached that level. Up to one week postoperatively, the proportion of eyes with an UCDVA of 0.7 had further increased to 89 % in the tPRK group and to 34 % in the PRK group. BCDVA was similar in both groups preoperatively (1.17 ± 0.18 in PRK group and 1.11 ± 0.15 in tPRK group; *p* = 0.082) as well as six weeks after surgery (1.21 ± 0.16 in PRK group and 1.18 ± 0.15 in tPRK group *p* = 0.128) and no patient had lost a line of preoperative BCDVA. The levels of accuracy of refractive correction were high regarding mean spherical equivalent off target at six weeks postoperatively. No statistically significant difference was observed between the two procedure groups or between right and left eyes (*p* > 0.05) (Fig. 3a-c). The achieved SE was within 1.0 D of the intended SE for all treated eyes and within ± 0.5 D for 84 % of eyes in the PRK group and within − 0.25 D and + 0.5 D for 82 % of eyes in the tPRK group

**Table 2:** Postoperative results

	PRK (*n*=100 eyes)			tPRK (*n*=100 eyes)			***P*** value*
	Right eyes	Left eyes	All eyes	Right eyes	Left eyes	All eyes	
**UCDVA** (decimal), mean ± SD							
Day 4	0.53 ± 0.09	0.53 ± 0.09	**0.53 ± 0.09**	0.73 ± 0.17	0.71 ± 0.17	**0.72 ± 0.17**	< 0.001
Day 7	0.62 ± 0.09	0.63 ± 0.08	**0.63 ± 0.08**	0.87 ± 0.19	0.84 ± 0.17	**0.86 ± 0.18**	< 0.001
6w	1.09 ± 0.07	1.1 ± 0.07	**1.1 ± 0.07**	1.1 ± 0.071	1.1 ± 0.07	**1.1 ± 0.07**	> 0.05
**BCDVA** (decimal), mean ± SD, 6w	1.21 ± 0.17	1.21 ± 0.16	**1.21 ± 0.16**	1.18 ± 0.15	1.18 ± 0.16	**1.18 ± 0.15**	> 0.05
**Refractive error (D),** mean ± SD, 6w							
Sphere	0.34 ± 0.28	0.30 ± 0.27	**0.32 ± 0.27**	0.22 ± 0.28	0.23 ± 0.27	**0.21 ± 0.30**	> 0.05
Cylinder	- 0.24 ± 0.13	- 0.25 ± 0.17	**- 0.23 ± 0.14**	-0.25 ± 0.26	-0.27 ± 0.32	**- 0.21 ± 0.21**	> 0.05
**SE off target**	0.13 ± 0.28	0.10 ± 0.28	**0.12 ± 0.28**	0.03 ± 0.21	0.03 ± 0.21	**0.03 ± 0.21**	> 0.05
**Pain Score (0-15),** mean ± SD	10.4 ± 1.37	11.16 ± 1.42	**10.8 ± 1.44**	5.80 ± 3.00	5.00 ± 2.70	**5.4 ± 2.84**	< 0.0001
**Epithelial healing (hours),** mean ± SD	60.2 ± 8.02	58.0 ± 5.15	**59.10 ± 6.79**	47.12 ± 9.22	44.40 ± 9.58	**45.76 ± 9.46**	< 0.0001

*BCDVA* best corrected distance visual acuity, *D* diopter, *UCDVA* uncorrected distance visual acuity, *PRK* photorefractive keratectomy, *tPRK* transepithelial photokeratectomy, *SE* spherical equivalent, *SD* standard deviation, *w* weeks

*p-value refers to comparison of PRK (all eyes) and tPRK (all eyes)

**Fig. 4:**
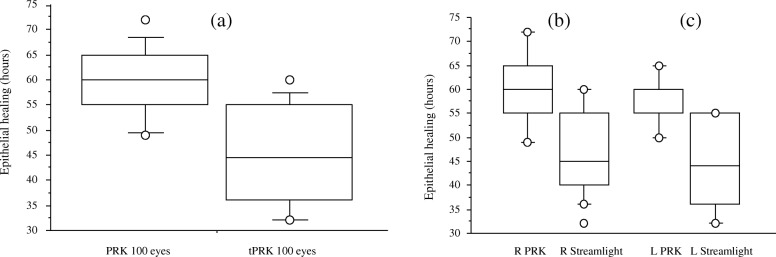
Time until complete epithelial wound healing in both treatment groups for (**a**) all eyes, **b** right eyes, **c** left eyes. Complete wound closure was achieved significantly faster in the tPRK group (*p* < 0.001)

**Fig. 5:**
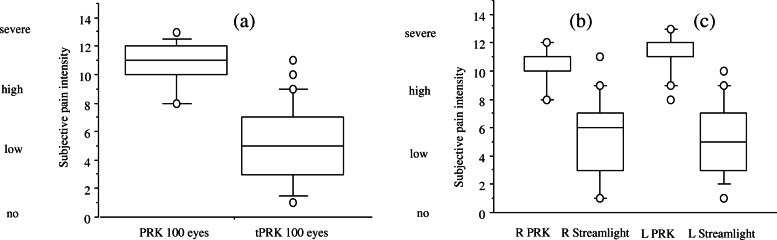
Subjective maximum pain intensity level within the first 4 days after surgery evaluated with a numeric pain rating scale in both treatment groups for (**a**) all eyes, **b** right eyes, **c** left eyes. Patients in the tPRK group experienced significantly less pain (*p* < 0.001)

Epithelial wound healing was achieved significantly faster in the tPRK group. Complete wound closure was observed in the tPRK group after in mean 45.76 ± 9.46 h, whereas it took in mean 59.10 ± 6.79 h in the PRK group (*p* < 0.0001) (Fig. 4a). No significant differences in re-epithelialization were observed between left and right eyes (Fig. 4b, c) nor between eyes with or without cylinder. Maximum pain level within the first 4 days after surgery revealed significantly less pain in the tPRK group compared to the PRK group (*p* < 0.0001) (Fig. 5a-c). No patient developed a postoperative corneal haze during the observation period and no other adverse effects or complications were observed.

## Discussion

To the best of my knowledge, this is the first study to evaluate the clinical results of StreamLight™ tPRK based on Alcon/Wavelight ablation profile in patients with mild to moderate myopia with and without mild astigmatism compared to PRK. The new StreamLight™ method requires only three parameters to calculate the WFO ablation profile: manifest refraction, optical zone and epithelial depth. This helps to reduce possible sources of error in the calculation of the ablation profile, especially in everyday hospital routine, and to implement efficient workflows and a very convenient calculation of the ablation profile.

The two patient groups examined in our study were comparable in terms of preoperative characteristics and reflect the typical patient population seeking refractive correction. The key findings of our study according to our early clinical results are that both treatments provide safe and effective refractive correction, but StreamLight™ tPRK is superior to PRK in terms of epithelial healing, postoperative pain and visual recovery time. This may considerably increase comfort with tPRK treatment.

The main reason for accelerated epithelial healing and reduced pain in the tPRK group could be the smaller epithelial defect. In PRK the epithelial removal zone is larger than the ablation zone, which might delay re-epithelialisation, whereas in StreamLight™, the size of the epithelial removal zone matches the size of the ablation zone. This might contribute to a faster re-epitheliialisation. In addition, also the PTK treated stromal bed may have provided the ideal basis for a fast and firmly adherent re-epithelialisation.

Although due to our daily busy clinic routine, it was not possible to observe wound closure hourly, all patients were instructed to come back to the clinic at the same time of day that the surgery took place on day 0. If a closed epithelium was noted on the slit lamp, the hours since surgery were counted. Despite the fact that this method does not record the exact time of epithelial closure, it can still be used for group comparison with regard to wound closure. Furthermore, an additional calculation of the chi-square test based on wound closure by days showed a significantly faster wound healing time in the Streamlight™ group (*p* < 0.0001). The shorter epithelial healing time after tPRK observed in our study has been shown in other clinical studies evaluating tPRK treatments performed with other nomograms and other laser systems. Fadlallah et al. observed an average period to complete healing of 2.5 days in the tPRK group versus 3.7 days in the PRK group while Naderi et al. reported 2.9 days and 3.3 days respectively [17, 19]. Although these results were obtained in different studies and therefore cannot be directly compared, it still has to be noted that in our study, complete closure of the epithelium after StreamLight™ tPRK was achieved faster, i.e., in less than two days.

This faster wound healing after tPRK may also have contributed to the fact that patients in the tPRK group perceived significantly less pain. Beyond that, however, pain and wound healing are closely linked and form a vicious circle [20]: in the presence of pain, more inflammatory parameters are released through the tear film and the conjunctiva, which is thought to slow down wound healing. In addition, pain contributes to patients squeezing and rubbing their eyes more often, which in turn mechanically impairs the newly forming epithelial cell layer. One of the most frequent complications after PRK is pain [14]. In order to minimize post-operative pain, our PRK procedure includes the avoidance of alcohol for epithelial removal, direct cooling of the cornea after ablation and the use of a bandage contact lens. Nevertheless, StreamLight™ tPRK patients still experienced significantly less pain during the early postoperative period compared to PRK patients. The median pain score of tPRK patients was 5.0, only half as high as the PRK patients’ median pain score of 11.0. This is in line with the results from other clinical studies on tPRK, indicating that postoperative pain after tPRK is lower compared to PRK [16, 17, 19].

According to our patients` feedback, one of the main reasons for most patients to opt for the StreamLight™ procedure, was the likelihood of faster wound healing and less pain, as well as the prospect of faster visual recovery. Although patients in both groups achieve a very good mean UCDVA of over 1.0 after 6 weeks, visual acuity in the tPRK group is significantly better from day 4. Almost three-quarters of all tPRK eyes but none of the PRK eyes achieved an UCDVA of 0.7 or better four days postoperatively. For patients, this is a crucial visual threshold, because permission for car driving is only given with a UCDVA of 0.7 or better in Germany [21]. Moreover, the rapid visual recovery after StreamLight™ tPRK can - combined with clever planning of the procedure close to the weekend - help to ensure that patients only have to take one day off work. Especially for the mostly young, employed patients this is another a very important aspect to choose this procedure.

Our study has limitations that should be addressed. First, the rate of epithelial healing was not objectively assessed by an image analysis software, nor was it possible to observe it hourly in our clinical routine. Secondly, the pain rating scale used was purely subjective. Moreover, a longer follow-up period is required to fully assess the development of visual acuity and refractive stability. However, the six-week follow-up period chosen in our study allows a proper evaluation visual recovery time, pain sensation and wound healing. In addition, our patients´ feedback on opting for the respective method might be biased by the fact that tPRK is offered in our practice at a price about 10 % higher than PRK. However, this can subliminally lead to both a better assessment of the postoperative results, but on the other hand also to a poorer assessment because the higher price might be associated with higher expectations of the postoperative results.

Overall, this study provides important insights from daily clinic routine with this novel tPRK method. According to our early clinical results, both methods appear to be safe and effective methods for the correction of low to moderate myopia, however, in our study, StreamLight™ tPRK offered faster visual recovery and epithelial healing compared to PRK, leading to less pain. It can therefore be considered a good treatment optionrequired to evaluate the efficac especially for patients who refuse or are not eligible for FS-Lasik, but at the same time demand a faster and more comfortable recovery time than PRK can offer. To confirm these results, further prospective, randomized studies with longer follow-ups are y and safety of this procedure in more detail.

## Data Availability

The datasets generated and/or analyzed during the current study are not publicly available due to institutional policy but are available from the corresponding author on reasonable request.

## Abbreviations

BCDVA: Best corrected distance visual acuity
D: Diopters
PRK: Photorefractive keratectomy
tPRK: Transepithelial photorefractive keratectomy
LASIK: Laser in situ keratomileusis
PVA: Polyvinyl acetal
SE: Spherical equivalent
UCDVA: Uncorrected distance visual acuity
